# Peer effects of health behaviors and the moderating role of Internet use among middle-aged and older adults: a nationally representative cross-sectional survey in China

**DOI:** 10.3389/fpubh.2024.1405675

**Published:** 2024-10-21

**Authors:** Yanyin Cui, Hongrui Bao, Fang Xia, Liya Zhang, Jie Ren

**Affiliations:** ^1^School of Humanities and Management, Zhejiang Chinese Medical University, Hangzhou, China; ^2^School of Health Management, Changchun University of Chinese Medicine, Changchun, China

**Keywords:** health behavior, middle-aged and older adult people, peer effects, Internet use, Internet preference

## Abstract

**Objective:**

This study investigates peer effects on the health behaviors of middle-aged and older adult people in China and examines the moderating role of Internet use.

**Methods:**

A total of 16,188 respondents were selected from the China Health and Retirement Longitudinal Study (CHARLS) 2020 data set. Ordinary Least Squares and Quantile Regression were used to validate the peer effect of health behaviors on middle-aged and older adults, and a moderating effects model was used to test the moderating roles of Internet use and Internet proficiency. Finally, the peer effect was analyzed for heterogeneity according to Internet use preferences.

**Results:**

The peer effect had a positive influence on the individual health behaviors of middle-aged and older adult people in China (*β* = 0.5341, *p* < 0.001). Middle-aged and older adult people were more sensitive to the average health behavior level of the community when they lived in rural communities and/or had a low level of education. Internet use positively moderated the health behavior peer effect in the older adult population (*β* = 0.0094, *p* < 0.1), and Internet proficiency negatively moderated the peer effect of the health behaviors of the middle-aged population (*β* = 0.1589, *p* < 0.1). According to the magnitude of the influence of peer effect, the Internet preference type can be ranked from social and entertainment (*β* = 0.6250, *p* < 0.01), to cultural and entertainment (*β* = 0.5835, *p* < 0.01), to comprehensive (*β* = 0.4622, p < 0.01).

**Conclusion:**

There is a peer effect on the health behaviors of middle-aged and old-aged individuals, in which Internet use plays a moderating role. The construction of healthy communities should be promoted, giving full play to the community’s active role in health promotion. Attention should be paid to the health-enabling function of the Internet, encouraging middle-aged and older adult people to use the Internet actively, moderately, and diversely, and making full use of the advantages of short videos for online health education.

## 1 Introduction

Population aging has become a global phenomenon, and China is one of the countries with the largest and fastest-aging older adult population in the world (1). By the end of 2021, China’s older adult population aged 65 years and over reached more than 200 million, accounting for 14.2% of the total population, at which point it formally transitioned from an “aging society” to an “aged society.” As a result, the health of middle-aged and older adult people has become a focus of social concern. Paying attention to the health behavior and lifestyle of the older adult contributes to the healthy aging of society as a whole and is one of the most important ways of actively coping with aging as a social issue (2). Health behaviors are an effective measure that is low-cost, highly prevalent, and can sustainably improve the health of the population (3). Good health behaviors can delay diseases or mitigate symptoms associated with aging in middle and old age, enhance physical functioning, and improve quality of life (4). Previous discussions on the factors influencing the health behaviors of the middle-aged and older adult have focused on individual factors such as age, education, gender, work, and income (5–7), or macro factors such as socio-economic and policy environments (8–11), while community factors, which lie in between the individual factors and the macro factors, have placed more emphasis on community building (12, 13) and neighborhood environments (14), and have not been presented as a concept of social relations under the concept of spatial constraints.

Due to China’s stronger cultural homogeneity and collectivist ideology, residents’ personal health behavior decisions are more likely to be influenced by others in the community (15). Scholars refer to the influence of group motivation on individual behavior as the peer effect, also known in economics texts as social capital, contagion, neighborhood effects, or peer group effects (16). Prior studies have confirmed that the peer effect has strong explanatory power with regard to residents’ fertility intentions, physical activity, and household consumption (17, 18), but there have been few studies of the peer effect of health behaviors on middle-aged and older adult groups. Merchant and others have found that peer-led projects to promote healthy aging in the community can reduce loneliness and improve the health of older people (19). Analyzing the influence of groups on individual health behaviors from a social interaction perspective is not only relevant to the health and well-being of the middle-aged and older adult but also an important reference for building a healthy aging society.

While the degree of population aging continues to deepen, modern digital technology continues to penetrate into the socio-economic life of the population and have a profound impact. The number of Internet users in China aged 60 and over has reached 119 million, accounting for 11.5% of the total number of Internet users, and the Internet penetration rate of the population aged 60 and over is 43.2% (20). More and more middle-aged and older adult people are conducting social, learning, entertainment, and consumption activities through the Internet, which promotes social participation and social integration and has an impact on health awareness and health literacy (21). The Digital Divide Theory suggests that differences in the way users make use of digital technology and their skill in using it can lead to individual differences in knowledge acquisition (22). For older people, being digitally included can help them maintain their independence, social connectedness, and sense of worth in the face of declining health or limited ability, while also providing new opportunities to improve their quality of life (23). Differences in Internet use among different groups of middle-aged and older adults also contribute to the issue of unequal access to health information (24). Many scholars have explored the correlation between overall Internet use and individual health behaviors in middle-aged and older adults, but the specific issues of Internet use proficiency and Internet use preference may also have an impact on individual health behaviors (25, 26). It is, therefore, necessary to study and understand the influence of Internet use proficiency and Internet use preference on the peer effect of health behaviors on middle-aged and older adult people.

Some researchers have explored the correlation between the Internet and health behaviors in middle-aged and older adults and have reported mixed results. However, there have been few studies to examine the mechanisms underlying the peer effect linking Internet use to health behaviors. Based on data from the 2020 China Health and Retirement Longitudinal Study (CHARLS), this study explores the peer effect and heterogeneity of health behaviors in middle-aged and older adult people, reveals the mechanism of Internet use in that effect, and investigates the differences between urban and rural areas, age groups, and usage preferences, to provide a basis for guiding the middle-aged and older adult to enhance their health behaviors and thereby improve their health. We will also study the differences between urban and rural areas in terms of age and usage preferences to provide a basis for guiding middle-aged and older adult people to improve their health behavior and health level.

## 2 Materials and methods

### 2.1 Data sources

The sample for this study was derived from the 2020 China Health and Retirement Longitudinal Study (CHARLS) dataset. The CHARLS sample covers 150 counties and districts, 450 villages and communities, randomly selected from across the country, with more than 10,000 households and approximately 18,000 individuals aged 45 years and older. It is, therefore, a good representation of China’s middle-aged and older adult population (27). In this study, those aged 45 and above were selected as the research subjects, and samples with missing key variables and samples with a community sample size of less than 20 were excluded, resulting in 16,188 valid observations.

### 2.2 Variable selection and description

Health behavior is the core explanatory variable of this study. Five indicators related to healthy living habits were selected, namely smoking, drinking, napping, exercising, and sleep duration, as were five indicators related to health risk prevention behaviors, namely outpatient behaviors, consumption of health care products, health check-ups, social pension insurance, and social medical insurance, all taken from the China Health and Retirement Longitudinal Study. The definitions of the specific indicators are given in Table 1. The study drew on the average Euclidean distance method used by Nan (28) to measure the accessibility of public health services, and the health behavior sub-indicators were summed to obtain a health behavior index for middle-aged and older adult people. First, the indices of healthy living habits and health risk prevention were summed separately, and then the two indices were synthesized into a total index of health behaviors. This method satisfies the characteristics of the indicator units being independent, bounded, and monotonic, thus effectively measuring the equilibrium within the same group.

**Table 1:** Health behavior indicators for middle-aged and older adult people.

Variables	Problem item	Answer
Healthy habits		
Smoking	Do you smoke, have you quit, or have you never smoked?	1 = *still smoke*; 2 = *quit*; 3 = *never smoked*
Drinking alcohol	In the past year, did you drink alcohol? How often did you drink?	1 = *Drink, more than once a month*; 2 = *Drink, but less than once a month*; 3 = *Drink nothing at all*
Napping	Over the past year, did you habitually nap?	1 = *yes*, 0 = *no*
Exercise (34)	Do you usually have at least ten minutes of sustained exercise activity (moderate or high intensity) per week?	1 = *yes*, 0 = *no*
Length of sleep (24)	During the past month, on average, about how many hours per night did you actually sleep?	1 = 6–9 *h*, 0 = o*ther*
Health risk prevention behaviors		
Outpatient behavior	In the past month, have you visited a healthcare facility for an outpatient visit or received home healthcare services?	1 = *yes*, 0 = *no*
Consumption of healthcare products	In the past month, were there any health care expenses including fitness exercise equipment, healthcare equipment and products, etc.?	1 = *yes*, 0 = *no*
Health check-ups	When was your last routine health check-up?	1 = *less than one year*; 0 = *more than one year without medical examination*
Social pension insurance	Are you covered by social pension insurance?	1 = *yes*, 0 = *no*
Social health insurance	Are you covered by social health insurance?	1 = *yes*, 0 = *no*

The explanatory variable in this study is the peer effect. Following the example of prior studies, this study set individuals living in the same community/village as belonging to the same peer group (29). The peer effect was calculated as the mean value of the level of health behaviors in the same community/village other than the respondent himself/herself. This study included three moderating variables: Internet use, Internet use proficiency, and Internet use preference. Internet use was measured by the item, “In the past month, did you go online?,” while Internet use proficiency was measured by the item, “Do you use a mobile phone to make payments, such as with Alipay, WeChat wallet, etc.?”; answers were coded as 1 = *yes*, 0 = *no*. Internet use preference was measured by the item, “What do you usually do online?,” with possible answers being chatting, watching the news, watching videos, playing games, and finance.

This study also controls for other variables that may influence an individual’s health behavior. These include age, age squared, gender, education level, community type, health level, household type, marital status, whether or not they are cohabiting, work status, and total annual income.

### 2.3 Research methodology

This study used Stata16 for statistical analysis of the data. The Ordinary Least Squares (OLS) model, Quantile Regression, and the Virtual Cohort Test (VCT) were used to analyze the influence of group effects on the health behaviors of middle-aged and older adult people. The interaction term model was used to test the moderating role of Internet use and Internet proficiency in the group effects of health behaviors on middle-aged and older adult people, and subgroup regression was used to test the heterogeneity of populations, communities, and Internet use preferences. The test level was *α* = 0.05.

## 3 Results

### 3.1 Descriptive statistics

A total of 16,188 middle-aged and older adult people were included in this study, of whom 7,696 (47.54%) were men and 8,492 (52.46%) were women. 7,479 (46.20%) were aged 45–59 years, and 8,709 (53.80%) were aged 60 years and above; 13,841 (85.50%) were with spouses, and 2,347 (14.50%) without; the community type was 10,733 (66.30%) rural and 5,455 (33.70%) urban; 12,440 (76.85%) lived in agricultural households, and 3,748 lived in non-agricultural households. With regard to educational level, 10,627 (65.65%) had completed primary school or below, 5,279 (32.61%) had completed secondary school, and 282 (1.74%) had attended tertiary education. With regard to working status, 11,045 (68.23%) were employed and 5,143 (31.77%) were either unemployed or retired.

### 3.2 Regression results for peer effects on the health behaviors of middle-aged and older adult people

Table 2 presents the estimation results from the Ordinary Least Squares (OLS) model and Quantile Regressions. The results of the OLS model show that the average level of health behavior of middle-aged and older adult people in the community other than the person himself had a significant effect on individual health behavior (*β* = 0.5341, *p* < 0.001). Specifically, for every 1-unit increase in the average health behavior level of the community, the health behavior of individual middle-aged and older adult people increased by 53.41 percentage points. Quantile regression was used to determine whether the peer effect for middle-aged and older adult people with different levels of health behaviors differed significantly. The estimated coefficients of the quantile regression can be interpreted as the marginal effects of the explanatory variables at specific quantile points; Table 2 presents the regression results at the three quantile points of 0.25, 0.5, and 0.75. The results show that the average level of health behavior of others in the community had a significant positive effect on individual health behavior, and that effect increases first and then decreases as the quantile points increase, with estimated coefficients of 0.4403, 0.4693, and 0.3985, in that order. This suggests that middle-aged and older adult people with average levels of health behaviors were more susceptible to the influence of the health behaviors of others, relative to the lower and higher groups.

**Table 2:** Results of least squares and quantile regression analyses of peer effects of health behaviors in middle-aged and older adults.

Variables	OLS regression		25% Quantile regression		50% Quantile regression		75% Quantile regression	
	Coef.	Std. Err.	Coef.	Std. Err.	Coef.	Std. Err.	Coef.	Std. Err.
Peer effect	0.5341^***^	0.0286	0.4403^***^	0.0484	0.4693^***^	0.0315	0.3985^***^	0.0377
Gender	−0.1887^***^	0.0021	−0.2408^***^	0.0017	−0.2005^***^	0.0022	−0.1565^***^	0.0028
Age	0.0043^***^	0.0012	0.0049^***^	0.0015	0.0030^**^	0.0012	0.0027^**^	0.0012
Age squared	−0.0001^***^	0.0001	−0.0001^***^	0.0001	−0.0001^*^	0.0001	−0.0001	0.0001
Marital status	−0.0265^***^	0.0029	−0.0254^***^	0.0044	−0.0245^***^	0.0043	−0.0187^***^	0.0031
Neighborhood type	−0.0025	0.0024	0.0004	0.0032	−0.0005	0.0025	−0.0058^**^	0.0026
Household type	−0.0026	0.0027	−0.0001	0.0038	−0.0059^*^	0.0031	−0.0041	0.0030
Educational attainment	0.0168^***^	0.0021	0.0139^***^	0.0034	0.0157^***^	0.0027	0.0148^***^	0.0032
Number of children	−0.0013	0.0008	−0.0030^***^	0.0010	−0.0016^***^	0.0006	−0.0002	0.0010
Work Status	0.0061^**^	0.0024	0.0012	0.0024	0.0076^***^	0.0025	0.0090^***^	0.0028
Total Annual Income	−0.0011^***^	0.0002	−0.0012^***^	0.0003	−0.0012^***^	0.0002	−0.0009^**^	0.0003
Health self-assessment	0.0042^***^	0.0009	0.0028^**^	0.0011	0.0031^***^	0.0010	0.0033^***^	0.0008
_cons	0.2317^***^	0.0440	0.2409^***^	0.0499	0.3314^***^	0.0463	0.4427^***^	0.0470
R2	0.364		0.2741		0.2296		0.1664	

***, **, and * denote significant at the 1, 5, and 10% confidence levels, respectively, as in the table below.

### 3.3 Robustness tests

#### 3.3.1 Replacing the explanatory variables

Considering the multidimensionality of health behaviors, this study repeated the regression using healthy living habits and health risk prevention ability as explanatory variables. As seen in Table 3, the average level of health behaviors of the middle-aged and older adult in the community had a significant positive impact on the healthy living habits (*β* = 0.5926, *p* < 0.001) and health risk prevention behaviors of the middle-aged and older adult individuals (*β* = 0.4568, *p* < 0.001), which is in line with the results of the benchmark regression. This indicates that the regression results of this study are relatively robust.

**Table 3:** Peer effect regression results for health behaviors of middle-aged and older adults (replacing explanatory variables).

Variables	Healthy lifestyle habits		Healthy risk prevention behaviors	
	Coef.	Std. Err.	Coef.	Std. Err.
Peer effect	0.5926^***^	0.0400	0.4568^***^	0.0340
Control variables	Control		Control	
_cons	0.4681^***^	0.0616	−0.2584^***^	0.0523
R2	0.3995		0.0628	

#### 3.3.2 Heterogeneity analysis

Although the results of the baseline regression indicated the existence of a peer effect on health behaviors, given the potential influence of differences in external characteristics, this study conducted a group regression from age, gender, neighborhood type, and educational attainment to further examine the peer effect of health behaviors on middle-aged and older adult people with different characteristics. Table 4 presents the regression results of the heterogeneity analysis.

- Age groups: The average level of community health behavior positively affected both middle-aged (*β* = 0.5422, *p* < 0.01) and older adult people (*β* = 0.5256, *p* < 0.01). There were no significant differences between these age groups.
- Gender: Both males (*β* = 0.5463, *p* < 0.01) and females (*β* = 0.5247, *p* < 0.01) experienced a significant positive effect from community health behavior. No significant differences were found between genders.
- Neighborhood Type: Significant positive effects were seen in both rural (*β* = 0.5824, *p* < 0.01) and urban (*β* = 0.4408, *p* < 0.01) communities. The effect was more pronounced in rural areas.
- Educational attainment: Significant positive effects were found for those with secondary school education (*β* = 0.5192, *p* < 0.01) and primary school or below (*β* = 0.5436, *p* < 0.01). For those with college education or above, the effect was non-significant (*β* = 0.2431, *p* > 0.1).

**Table 4:** Results of peer effect heterogeneity analysis of health behaviors in middle-aged and older adults.

Variables	Heterogeneity grouping	Sample size	Coef.	Std. Err.
Age	Middle-aged (45–59 years old)	7,479	0.5422***	0.0421
	Older adult (≥60 years old)	8,709	0.5256***	0.0388
Gender	Men	7,696	0.5463***	0.0481
	Women	8,492	0.5247***	0.0323
Neighborhood type	Rural community	10,733	0.5824***	0.0355
	Town community	5,455	0.4408***	0.0485
Educational attainment	Primary and below	10,627	0.5436***	0.0343
	Secondary school	5,279	0.5192***	0.0527
	College and above	282	0.2431	0.2126

### 3.4 Analysis of the moderating roles of Internet use and Internet proficiency

This section concerns the moderating effects of Internet use and Internet proficiency on the peer effect of health behaviors on middle-aged and older adults and the related population heterogeneity. By adding the cross-multiplier terms of Internet use and peer effect, the Ordinary Least Squares model was used to regress the sample of middle-aged and older adults, the sample of rural and urban communities, and the total sample. The results are shown in Table 5.

- Total sample: Internet use had a significant promotional effect on personal health behaviors (*β* = 0.0100, *p* < 0.01). The interaction term for Internet use and peer effects had a positive but insignificant effect in the total sample (*β* = 0.0323, *p* > 0.1).
- Neighborhood type: The effect of Internet use on personal health behaviors had significant positive effects in both urban (*β* = 0.0111, *p* < 0.01) and rural (*β* = 0.0094, *p* < 0.01) communities. The interaction term for Internet use and peer effects had a positive but insignificant effect, both in the rural community subgroup (*β* = 0.0810, *p* > 0.1) and the urban community subgroup (*β* = 0.0425, *p* > 0.1).
- Age groups: The effect of Internet use on personal health behaviors was significant in both older people (*β* = 0.0128, *p* < 0.01) and middle-aged people (*β* = 0.0094, *p* < 0.05). The effect was more pronounced in older people. The interaction term had a negative (*β* = −0.0842, *p* > 0.1) but insignificant effect in the middle-aged group. However, its effect was significantly positive (*β* = 0.0094, *p* < 0.1) in the older adult population.

**Table 5:** Moderating effects of Internet use and analysis of population and community heterogeneity.

Variable	Total		Middle-aged		Older adult		Rural community		Urban community	
	Coef.	SE.	Coef.	SE.	Coef.	SE.	Coef.	SE.	Coef.	SE.
Peer effect	0.5310^***^	0.0286	0.5620^***^	0.0479	0.5498^***^	0.0421	0.5870^***^	0.0360	0.4277^***^	0.0515
Internet usage	0.0100^***^	0.0024	0.0080^**^	0.0031	0.0128^***^	0.0037	0.0094^***^	0.0030	0.0111^***^	0.0041
Internet usage*Peer effect	0.0323	0.0574	−0.0842	0.0889	0.1539^*^	0.0912	0.0810	0.0754	0.0425	0.0971
Control variables	Control		Control		Control		Control		Control	
_cons	0.2031^***^	0.0445	0.4696^*^	0.2845	−0.1963	0.1406	0.1434^***^	0.0540	0.3286^***^	0.0812
Sample size	16,188		7,479		8,709		10,733		5,455	
R2	0.3648		0.4234		0.3170		0.3685		0.3579	

This indicates that Internet use positively moderated the health behavior peer effect in the older adult population.

Table 6 presents the results of the moderating effects test and the population and community heterogeneity analysis for Internet proficiency.

- Total sample: Internet proficiency had a significant facilitating effect on personal health behaviors (*β* = 0.0106, *p* < 0.01). The interaction term of Internet proficiency and peer effect had a negative but statistically insignificant effect in the total sample (*β* = −0.0584, *p* > 0.1).
- Neighborhood type: The effect of Internet proficiency on personal health behaviors was significantly positive in both urban (*β* = 0.0118, *p* < 0.01) and rural (*β* = 0.0096, *p* < 0.01) communities. The interaction term of Internet proficiency and peer effect had a negative but insignificant effect both in the rural (*β* = −0.0219, *p* > 0.1) and urban (*β* = −0.0352, *p* > 0.1) community subgroups.
- Age groups: The effect of Internet proficiency on personal health behaviors was significantly positive in both the older adult (*β* = 0.0117, *p* < 0.05) and middle-aged (*β* = 0.0103, *p* < 0.01). The effect was higher among the older adult. The interaction term had a positive but insignificant effect in the older adult population (*β* = 0.1412, *p* > 0.1), and a significant negative effect in the middle-aged population (*β* = −0.1589, *p* < 0.1).
- This suggests that Internet proficiency negatively moderated the peer effect of health behaviors in the middle-aged population.

**Table 6:** Moderating effect of Internet proficiency and analysis of population and community heterogeneity.

Variable	Total		Middle-aged		Older adults		Rural community		Urban community	
	Coef.	SE.	Coef.	SE.	Coef.	SE.	Coef.	SE.	Coef.	SE.
Peer effect	0.5305^***^	0.0286	0.5715^***^	0.0462	0.5449^***^	0.0439	0.5780^***^	0.0360	0.4373^***^	0.0508
Internet proficiency	0.0106^***^	0.0027	0.0103^***^	0.0032	0.0117^**^	0.0053	0.0096^***^	0.0036	0.0118^***^	0.0043
Internet proficiency*Peer effect	−0.0584	0.0644	−0.1589^*^	0.0840	0.1412	0.1317	−0.0219	0.0918	−0.0352	0.0976
Control variables	Control		Control		Control		Control		Control	
_cons	0.1982^***^	0.0448	0.4401	0.2843	−0.1772	0.1408	0.1513^***^	0.0541	0.3028^***^	0.0818
Sample size	16,188		7,479		8,709		10,733		5,455	
R2	0.3647		0.4238		0.3163		0.3683		0.3579	

### 3.5 Heterogeneity analysis of Internet use preferences

Internet usage preferences were clustered into three groups based on five activities—chatting, watching news, watching videos, playing games, and managing money—measured by the question, “What do you usually do online?” These five activities were K-means clustered into three groups: cultural and entertainment (watching videos, watching news, playing games, and managing money), social and entertainment (chatting and watching videos), and comprehensive (chatting, watching videos, watching news, playing games, and managing money). Among the participants, there were 2,754 middle-aged and older adult people whose Internet use preferences were cultural and entertainment, accounting for 41.73% of the total number of people using the Internet; 1,239 people, or 18.77%, had a preference for social and entertainment; and 2,607 people, or 39.50%, were assigned to the comprehensive group. Table 7 presents the regression results for the peer effect of health behaviors on middle-aged and older adult people with different types of Internet use preferences. The results show that the size of the influence of the peer effect on those with different Internet use preferences was in the order of social-entertainment type (*β* = 0.6250, *p* < 0.01), cultural-entertainment type (*β* = 0.5835, *p* < 0.01), and comprehensive type (*β* = 0.4622, *p* < 0.01).

**Table 7:** Heterogeneity analysis of Internet use preferences.

Variable	Cultural and entertainment		Social and entertainment		Comprehensive	
	Coef.	SE.	Coef.	SE.	Coef.	SE.
Peer effect	0.5835^***^	0.0753	0.6250^***^	0.0964	0.4622^***^	0.0677
Gender	−0.1914^***^	0.0055	−0.2058^***^	0.0080	−0.2030^***^	0.0052
Age	0.0016	0.0048	0.0025	0.0065	−0.0057	0.0045
Age squared	0.0001	0.0001	−0.0001	0.0001	0.0001	0.0001
Marital status	−0.0205^**^	0.0102	−0.0197^*^	0.0114	−0.0166^*^	0.0092
Peer effect	−0.0018	0.0063	0.0132	0.0089	0.0035	0.0060
Educational attainment	0.0226^***^	0.0049	0.0006	0.0073	0.0208^***^	0.0048
Number of children	0.0007	0.0025	−0.0007	0.0033	−0.0001	0.0025
Work status	0.0102	0.0067	0.0096	0.0086	−0.0128^*^	0.0066
Total annual income	−0.0007	0.0006	−0.0010	0.0008	−0.0004	0.0006
Health self-assessment	0.0056^**^	0.0026	0.0002	0.0033	−0.0015	0.0026
_cons	0.2374	0.1513	0.2461	0.1972	0.5792^***^	0.1423
Sample size	2,754		1,239		2,607	
R2	0.3315		0.3815		0.4076	

## 4 Discussion

### 4.1 Positive influence of peer effects on health behaviors in middle-aged and older adults

The average level of health behaviors of middle-aged and older adult people in the community was positively correlated with individual health behaviors, suggesting that there is a peer effect on the health behaviors of middle-aged and older adult people. This result is consistent with the findings of Xie Donghong et al. (15), who found that an improvement in the average level of health in the community promotes proactive health-seeking behaviors. According to the theory of the peer effect, the performance of specific behaviors by an individual is often influenced by the behaviors and characteristics of other individuals in their social group, leading to similarities with the group’s behavioral performance (30); health behaviors are one type of such behaviors that are influenced by the surrounding group. The community is the main place where residents conduct their daily activities and an important unit for the peer effect (31). The peer effect of health behaviors on middle-aged and older adult people may exert its effects through the following three mechanisms.

The first is the social interaction mechanism: social interactions may change an individual’s perceptions and ideas about health, and even influence their judgments about health behaviors. The older adult in a community can form a relational social network based on trust and reciprocity, and, in the process of social interaction, they will pass on and exchange healthcare information, thus guiding other individuals in making decisions regarding their health behaviors (32).

The second is a culture-driven mechanism: due to the collectivist mindset that is dominant in the older group, the middle-aged and older groups are more proactive in seeking out healthy behaviors and aligning themselves with group behaviors when there is an increase in the overall level of health behaviors in the community (33). This peer effect of health behaviors occurs through interactions between neighbors, and the stronger the interactions between middle-aged and older adult people in the community, the greater the influence on individuals. Traditional Chinese neighborhood culture emphasizes the idea that neighbors are good companions. The frequent interactions among neighbors in the community under the acquaintance society promote the flow of health information.

The third is the social norms mechanism: based on social rules, people subconsciously look to the dominant behavioral norms as a guide to their own behavior in a given social situation (34). The Outline of the “Healthy China 2023” Plan, under the theme of “building and sharing health for all,” makes the building of healthy communities an important part of the construction of a healthy China. Each community promotes healthy behaviors and discourages unhealthy behaviors by optimizing their social environment and formulating relevant systems such as the Healthy Community Rules and System Measures, thereby raising the average level of healthy behaviors in the middle-aged and older adult people in the community. The health disparity utility of communities and groups reinforces each other, and the community, as an important unit of peer effect, can be a reliable vehicle for health messaging and health policy implementation. Therefore, the government should give full play to the dynamic role of the community, encourage its guidance and support for community health promotion work, and promote the building of a healthy community.

In summary, there is a peer effect on the health behaviors of middle-aged and older adult people in a community in the context of Chinese collectivist culture. The results of this study are informative for the formulation of guidance for community residents’ health behaviors in collectivist-based social structures. In addition, the results of this study provide lessons relevant to the health level of immigrant communities in an individualism-based social structure. Immigrant communities are often culturally homogeneous and foster frequent communication and interaction, facilitating peer phenomena. Enhancing the health of others through the influence of group members can have cost-effective effects and impacts that go far beyond the initial design conceptualization.

### 4.2 Peer effects of health behaviors on middle-aged and older people differ by area of residence and level of education

The peer effect of health behaviors on middle-aged and older adult people is heterogeneous in terms of area of residence and level of education. Compared to urban communities, the health behaviors of middle-aged and older people in rural communities are more likely to be influenced by the average level of health behaviors in their community. Rural areas are natural communities, less subject to residential cluster effects and more conducive to the identification of health behavioral peer effects in middle-aged and older people (29). In addition, those who live in the countryside are more likely to form a society of acquaintances in which there are strong interactions and interdependence among middle-aged and older individuals, which reinforces the dissemination of health information (35). In terms of education level, the peer effect of health behaviors was the most effective in promoting health behaviors in middle-aged and older adult people with primary school education and below, slightly higher than that in the secondary school education group. However, there was no peer effect on the health behaviors of middle-aged and older adult people who had a tertiary education or above. There are three possible reasons for this. First, the middle-aged and the older adult with a lower level of education have fewer channels to obtain health information; the weaker their level of behavioral decision-making and judgment, the more likely that they will be influenced by role models in the process of social interaction, and the more sensitive they will be to the peer effect of health behaviors. Second, people with a high level of education usually have stronger cognitive and information comprehension and analytical and processing abilities, and are able to acquire health-related knowledge from multiple sources, resulting in a higher level of health literacy (36). As a result, their health behaviors are more autonomous in terms of decision-making and less affected by the peer effect of health behaviors. From the perspective of policymakers, rural communities and middle-aged and older groups with low levels of education should receive more policy-oriented support and assistance. For example, the role of health education should be brought into play to advocate for the adoption of positive health behaviors and lifestyles by the middle-aged and older adult; policy advocacy can be tailored toward specific groups of people, guiding cadres, party members, or opinion leaders with a high level of literacy to play the role of exemplars of health behaviors and give full play to the demonstrative effect of health behaviors.

### 4.3 Internet use and Internet proficiency were moderators in older and middle-aged populations, respectively

Internet use played a significant and positive moderating role in the peer effect of health behaviors in the older population, i.e., Internet use increased the average health behaviors of older individuals. First, older persons have withdrawn from the labor market, with the consequent loss of the formal social roles and social relationships associated with work, so that their social networks are ultimately confined to their family and friends. Second, as physical function declines, so does the frequency of socializing and social activities among older people. Older adults who use the Internet extend their social network while staying in close contact with family and friends (37, 38). The stronger the social interaction, the more pronounced the peer effect of health behaviors. In addition, in the current context of widespread smartphone usage in China, the older adult can independently engage in searching for medical care and obtaining health information through the use of smartphones. Internet use provides a degree of independence for older people, freeing them from having to rely entirely on their children for personal care and attention (39). At the same time, Internet use helps older people obtain a wealth of health information and improve their disease prevention awareness; the resulting health literacy can help them develop good health behaviors (40). However, the spread of social media has also led to the proliferation of false health information. Intergenerational support from children can help older adults better use online health information, thus bridging the digital divide and promoting healthy behaviors (39, 41).

Meanwhile, our results also show that Internet proficiency played a significant negative moderating role in the peer effect of health behaviors on the middle-aged population, i.e., Internet proficiency reduced the impact of health behaviors on middle-aged individuals. There are two possible reasons for this. On the one hand, it has been argued that using devices to access information is a basic skill, and that the ability to recognize the value of information and quickly access needed information is particularly important (42). Unlike older people, middle-aged people have richer types of Internet online activities and more complex social networks, are able to distinguish and absorb information from different information channels, and are less likely to be influenced by a single group. On the other hand, the proficiency of middle-aged and older adults at Internet use implies that Internet addiction may be a problem (43). Substitution effects theory suggests that the time middle-aged people spend in online communities erodes their physical community interactions, which may diminish the impact of the peer effect. Therefore, in the process of continuing to improve the popularity of the Internet, the relevant departments (e.g., government agencies such as the Information and Communication Authority (ICMA), academic organizations such as Population Development and Gerontology Management (PDGM), age-friendly smart product development organizations, etc.) should provide guidance on reasonable use of the Internet for the middle-aged and older adult and give full play to the positive role of the Internet. For middle-aged adults living in the community, providing access to and training in digital technology can increase their self-efficacy with digital devices, thereby increasing the frequency of effective Internet-based communication with friends and family and reducing social isolation (44). At the same time, it is also necessary to make good use of the Internet as a carrier of health information and to enhance the older adult’s awareness of their health and their ability to take an active role in their own health through the transmission of health knowledge and concepts.

### 4.4 Peer effects of health behaviors on middle-aged and older adults present heterogeneity in terms of Internet use preferences

The peer effects of health behaviors on middle-aged and older adult people differed in terms of Internet use preferences. The peer effect of health behaviors on middle-aged and older adult people whose Internet preference was social and recreational was higher than that on those whose Internet preference was cultural and recreational, and the peer effect for those whose preference was cultural and recreational was higher than that for those whose preference was comprehensive, i.e., the peer effect of health behaviors was more sensitive among middle-aged and older adult people who used the Internet socially and instrumentally (i.e., for obtaining information, etc.).

First, middle-aged and older adult people whose preference is social entertainment go online mainly to watch videos and chat, and through chatting and short videos they enhance their interaction with friends and relatives and maintain social relationships (45). Short videos with social functions are simple to upload and rich in information, and thus can help middle-aged and older adult people share their lives and pay attention to the health conditions of their friends and relatives. Liking, commenting, forwarding, and interacting with each other on short video platforms can make up for the lack of offline communication, making the social distance between individuals closer and the peer effect of health behaviors stronger.

Second, middle-aged and older adult people with a cultural and recreational focus have active learning and self-entertainment online behaviors, mainly consisting of watching videos, reading news, playing games, and managing money. The Internet has the function of mass communication and interpersonal communication, making it possible for middle-aged and older adult people to learn a wealth of health knowledge through the Internet. The result is that the Internet changes the health behavior of the older adult through persuasive communication (46), and the peer effect is more likely to have an impact through the social learning and cohort mechanisms. It is worth noting that Internet videos have an advantage in Internet active learning among middle-aged and older adult people, in that, compared with static information, short videos are more vivid and intuitive, and are therefore more likely to be accepted by middle-aged and older adult people who have a low cultural level and limited reading ability (47).

Finally, the peer effect of health behaviors on middle-aged and older adult people whose Internet preference was of the comprehensive type was relatively low. The results of this study show that the proportion of middle-aged and older adult people with high Internet proficiency whose Internet preference was comprehensive was 46.99%, and their online activities included chatting, watching videos, reading news, playing games, and managing money. This indicates that middle-aged and older adult people with a comprehensive preference have rich online activities and high Internet networking proficiency and frequency of use.

There is some degree of Internet addiction and Internet fraud afflicting middle-aged and older adult people (48). The physiological functions of middle-aged and older adult people are in decline, and excessive use of the Internet will consume a certain amount of their energy and physical strength, but it is not conducive to the promotion of healthy behaviors, such that the peer effect is relatively weakened. This suggests that we should take advantage of the rapid development of the Internet and short videos as a form of communication in promoting health behavior change among the middle-aged and older adult. Older adults have lower cyber-efficacy and digital literacy, and thus they are less able to identify and defend against false health information. Under the Chinese culture of filial piety, intergenerational relationships can serve as a safety net for the older adult (41). High-quality intergenerational relationships can reduce the incidence of healthcare fraud among the older adult. There is a need to effectively address the problem of Internet fraud among the middle-aged and older adult by increasing publicity efforts to encourage children to pay attention to the emotional needs of their parents and increase the frequency of emotional communication with them (49).

### 4.5 Limitations

Despite these important findings, this study has some limitations. First, it focuses on middle-aged and older adult individuals in the community under the Chinese socio-cultural context. Its results may not be generalizable to a wider population or a different economic and cultural environment. Second, the cross-sectional data limit our ability to draw any causal inferences about the impact of Internet use on the heterogeneity of peer effects on health behaviors. Third, the two dimensions of health behavior were based on a collation of indicators from the database. Therefore, the health behavior profile of respondents used in this study may not be comprehensive. Fourth, only three variables related to Internet use (namely Internet use, Internet proficiency, and Internet preference) were used due to the limitations of the available data. Further studies should consider adding other explanatory variables such as type, kind, and duration of Internet use. Finally, the data may be outdated because the latest round of CHARLS was updated in 2020.

## 5 Conclusion

Based on a national sample of middle-aged and older adult Chinese people, this study verified the peer effect of health behaviors and the mechanisms by which Internet use affects them. This study revealed that the average health behavior level of middle-aged and older adult people in the community positively promotes individual health behaviors, and that middle-aged and older adult people in rural communities and with primary school education or below are more likely to be affected by the peer effect of health behavior. In addition, we found that Internet use was a positive moderator in older adults and Internet proficiency was a negative moderator in the middle-aged. The peer effects of health behaviors on middle-aged and older adults differed with their Internet use preferences. In summary, this study provides new empirical evidence for the relationship between Internet use and health behavior in middle-aged and older adults, and enriches the literature on health promotion in middle-aged and older adults in the Internet era.

## Data Availability

Publicly available datasets were analyzed in this study. This data can be found at: https://charls.pku.edu.cn/.

## References

[1] Ai Z Tang C Wen X Kartheepan K Tang S. Examining the impact of chronic diseases on activities of daily living of middle-aged and older adults aged 45 years and above in China: a nationally representative cohort study. Front Public Health. (2024) 11:1303137. doi: 10.3389/fpubh.2023.1303137, PMID: 38419813 PMC10899675

[2] WHO. *Ageing Health*. (2022). Available at: https://www.who.int/news-room/fact-sheets/detail/ageing-and-health (Accessed March 5, 2023)

[3] Cawley J Ruhm CJ. The economics of risky health behaviors. Handbook Health Econ. (2011) 2:95–199. doi: 10.1016/B978-0-444-53592-4.00003-7

[4] Ramage-Morin PL Shields M Martel L. Health-promoting factors and good health among Canadians in mid- to late life. Health Rep. (2010) 21:45–53. PMID: 20973433

[5] Smith ML Colwell B Ahn S Ory MG. Factors associated with tobacco smoking practices among middle-aged and older women in Texas. J Women Aging. (2012) 24:3–22. doi: 10.1080/08952841.2012.638876, PMID: 22256875

[6] Liu L Zhang Y Wu W Cheng R. Characteristics of dental care-seeking behavior and related sociodemographic factors in a middle-aged and elderly population in Northeast China. BMC Oral Health. (2015) 15:66. doi: 10.1186/s12903-015-0053-3, PMID: 26070786 PMC4465149

[7] Hooker SP Wilcox S Burroughs EL Rheaume CE Courtenay W. The potential influence of masculine identity on health-improving behavior in midlife and older African American men. J Mens Health. (2012) 9:79–88. doi: 10.1016/j.jomh.2012.02.001, PMID: 23459337 PMC3580856

[8] Han TS Lee DM Lean ME Finn JD O'Neill TW Bartfai G. Associations of obesity with socioeconomic and lifestyle factors in middle-aged and elderly men: European male aging study (EMAS). Eur J Endocrinol. (2015) 172:59–67. doi: 10.1530/EJE-14-0739, PMID: 25326134

[9] Stamatakis E Grunseit AC Coombs N Ding D Chau JY Phongsavan P. Associations between socio-economic position and sedentary behaviour in a large population sample of Australian middle and older-aged adults: the social, economic, and environmental factor (SEEF) study. Prev Med. (2014) 63:72–80. doi: 10.1016/j.ypmed.2014.03.009 24650626

[10] Lu J Liu L Wang Y Zhou Z. Social engagement and urban-rural disparity in self-management behaviors: study of middle-aged and dlder Chinese hypertension patients. Front Public Health. (2022) 9:801307. doi: 10.3389/fpubh.2021.801307, PMID: 35155352 PMC8828651

[11] Lan L Hai P Luo J Li R Wang Y. Medical behaviours and medication adherence of older hypertensive patients in different medical insurance programs in Beijing, China: a cross-sectional study. BMC Geriatr. (2023) 23:878. doi: 10.1186/s12877-023-04476-y, PMID: 38124122 PMC10734068

[12] Mukherjee D Saxon V. "psychological boarding" and community-based behavioral health crisis stabilization. Community Ment Health J. (2019) 55:375–84. doi: 10.1007/s10597-018-0237-9 29380094

[13] McNeish R Rigg KK Tran Q Hodges S. Community-based behavioral health interventions: developing strong community partnerships. Eval Program Plann. (2019) 73:111–5. doi: 10.1016/j.evalprogplan.2018.12.005, PMID: 30580000

[14] Bayly JE Panigrahi A Rodriquez EJ Gallo LC Perreira KM Talavera GA. Perceived neighborhood factors, health behaviors, and related outcomes in the hispanic community health study/study of latinos. Prev Med. (2022) 164:107267. doi: 10.1016/j.ypmed.2022.107267, PMID: 36150447 PMC9691577

[15] Xie DH Zhu ZS. Study on the peer effect of health and its mechanism. S Chin Popul. (2020) 35:39–51.

[16] Ding W Lehrer SF. Do peers affect student achievement in China's secondary schools? Rev Econ Stat. (2006) 89:300–12. doi: 10.1162/rest.89.2.300

[17] Zhang KA. Study on the influence of the same group effect on household consumption expenditure: empirical evidence from CFPS2018 survey data. J Commercial Econ. (2023) 18:65–8.

[18] Xu WX Guo KL. Peer effect of physical exercise among community residents in China—an empirical analysis based on data from the chinese general social survey. Chin Sport Sci Technol. (2024):1–7. doi: 10.16470/j.csst.2023070

[19] Merchant RA Tsoi CT Tan WM Lau W Sandrasageran S Arai H. Community-based peer-led intervention for healthy ageing and evaluation of the ‘HAPPY’ program. J Nutr Health Aging. (2021) 25:520–7. doi: 10.1007/s12603-021-1606-6, PMID: 33786571 PMC7883995

[20] China Internet Network Information Center. The 49th statistical report on China’s internet development. (2020). Available at: https://www.cnnic.cn/NMediaFile/old_attach/P020220721404263787858.pdf (Accessed March 8, 2024).

[21] Yuan C Wei XM Wu XY Liu HL Jiang ZM. Habits of using online health information and e health literacy in middle-aged and Ecery residents. Chin Gen Pract. (2023) 26:1989–94.

[22] Lu JH Wei XD. Analysis framework,concept,and pathways of digital divide governance for olderadults:from the perspective of digital divide and knowledge gap theory. Popul Res. (2021) 45:17–30.

[23] Olphert W Damodaran L. Older people and digital disengagement: a fourth digital divide? Gerontology. (2013) 59:564–70. doi: 10.1159/000353630, PMID: 23969758

[24] Chang WL Li JX Ni WG XiaoY WZY Wang C Yang CX. Correlation between health information literacy and behavior habits among middle.Aged and elderly residents in community. Mod Prev Med. (2023) 50:2613–9.

[25] Sheng X Simpson PM. Seniors, health information, and the internet: motivation, ability, and internet knowledge. Cyberpsychol Behav Soc Netw. (2013) 16:740–6. doi: 10.1089/cyber.2012.0642, PMID: 23679569

[26] Nan Y Xie Y Hu Y. Internet use and depression among Chinese older adults: the mediating effect of interpersonal relationship. Front Public Health. (2023) 11:1102773. doi: 10.3389/fpubh.2023.1102773, PMID: 36935716 PMC10022907

[27] Zhao Y Yisong H Smith JP Strauss J Yang G. Cohort profile: the China health and retirement longitudinal study (CHARLS). Int J Epidemiol. (2014) 43:61–8. doi: 10.1093/ije/dys203, PMID: 23243115 PMC3937970

[28] Zhang N Gao MY Kou X. Cultural barriers to health equity —does cross-dialect migration reduce access to public health services? Finance Tr Econ. (2021) 42:36–50. doi: 10.19795/j.cnki.cn11-1166/f.20210207.009

[29] Xu J Wang YQ. Citizen volunteering in the context of common wealth research on cohort effect: empirical analyses based on CSS2019 and CSS2021. Soc Sci Hunan. (2023) 4:102–11.

[30] Liu C Zhang ZL. Peer effects and health status of middle-aged and older adults in rural China. S Chin Popul. (2022) 37:66–80+37.

[31] Hu XZ Gao HX Zheng PY. Community peer effect of healthcare consumption and moderating effect of social activities among middle-aged and elderly people in China. Med Soc. (2023) 36:53–8.

[32] Ferrer R Klein WM. Risk perceptions and health behavior. Curr Opin Psychol. (2015) 5:85–9. doi: 10.1016/j.copsyc.2015.03.012, PMID: 26258160 PMC4525709

[33] He Y She C Wang YP. Research on the effect of social interaction on the consumption upgrading of the elderly: on the economic value of square dance. J Financ Econ. (2021) 47:124–38. doi: 10.16538/j.cnki.jfe.20210217.401.

[34] Zhao WH Li KJ Wang YY. Physical activity guidelines for chinese (2021). Chin J Public Health. (2022) 38:129–30.

[35] Xu TB Chen X. Interactive mobility: health communication among the rural left-behind elderly--a case study of Yangzheng Village, Anhui Province, China. Youth Journal. (2023) 8:65–7. doi: 10.15997/j.cnki.qnjz.2023.08.008

[36] Xiao Q Su P Huang PZ. Correlation between health information literacy and behavior habits among middle.Aged and elderly residents in community. Chin J Health Educ. (2023) 39:1039–45.

[37] Zickuhr K Madden M. Older adults and internet use:for the first time, half of adults ages 65 and older are online. Pew Res Center’s Internet Am Life Proj. (2012)

[38] Zhou JJ Bai X. Influence of intergenerational relationships on depressive symptoms in ageing Chinese adults in Hong Kong: mediating effects of sense of loneliness. BMC Geriatr. (2022) 22:587. doi: 10.1186/s12877-022-03269-z, PMID: 35840878 PMC9287879

[39] Huang R Gong R Deng Q Hu Y. The effect of intergenerational support from children on loneliness among older adults-the moderating effect of internet usage and intergenerational distance. Front Public Health. (2024) 12:1330617. doi: 10.3389/fpubh.2024.1330617, PMID: 38655528 PMC11036867

[40] Aggarwal B Xiong Q Schroeder-Butterfill E. Impact of the use of the internet on quality of life in older adults:re-view of literature. Prim Health Care Res. (2020) 21:e55. doi: 10.1017/S1463423620000584, PMID: 33263273 PMC7737194

[41] Fu YY Ji XW. Intergenerational relationships and depressive symptoms among older adults in urban China: the roles of loneliness and insomnia symptoms. Health Soc Care Community. (2020) J28:131. 10.1111/hsc.12964 32115795

[42] Lyu S Sun J. Internet use and self-rated health among Chinese older adults: the mediating role of social capital. Geriatr Gerontol Int. (2021) 21:34–8. doi: 10.1111/ggi.14090, PMID: 33280230

[43] Jia Y Liu TY Yang Y. Trapped in the smartphone: intergenerational relationships and internet addiction among older adults. Journalism Res. (2023) 10:31–121. doi: 10.20050/j.cnki.xwdx.2023.10.006

[44] Phang JK Kwan YH Yoon S Goh H Yee WQ Tan CS. Digital intergenerational program to reduce loneliness and social isolation among older adults: realist review. JMIR Aging. (2023) 6:e39848. doi: 10.2196/39848, PMID: 36598801 PMC9850285

[45] Zhang C Xiang J. The impact of short video on the quality of life of older adults - based on social network perspective. J Xi'an Jiaotong Univ. (2024):1–15.

[46] Liu Y. Using the internet to change health behavior. J Huazhong Univ Sci Technol. (2008) 5:109–13. doi: 10.19648/j.cnki.jhustss1980.2008.05.020.

[47] Rus HM Camron LD. Health communication in social media: message features predicting user engagement on diabetes-related Facebook pages. Ann Behav Med. (2016) 50:678–89. doi: 10.1007/s12160-016-9793-9, PMID: 27059761

[48] Zhang Y Guo Y Li M. The moderating effect of intergenerational relationships on the association between internet engagement and mental well-being. Aging Ment Health. (2024) 28:36–44. doi: 10.1080/13607863.2023.2207479, PMID: 37139965

[49] Sharifi S Babaei Khorzoughi K Arch RM Geriatr G. The association between intergenerational relationships and depression among older adults: a comprehensive systematic literature review. Arch Gerontol Geriatr. (2024) 119:105313. doi: 10.1016/j.archger.2023.105313, PMID: 38101113

